# Intimate Partner Violence and Mental Health

**DOI:** 10.3402/ejpt.v5.25580

**Published:** 2014-09-12

**Authors:** Sheila Sprague, Miranda Olff

**Affiliations:** 1 Division of Orthopaedic Surgery, Department of Surgery, McMaster University, Hamilton, Ontario, Canada; 2 Department of Psychiatry, Academic Medical Centre, University of Amsterdam & Arq Psychotrauma Expert Group, Diemen, the Netherlands

Welcome to this special issue of the *European Journal of Psychotraumatology* on intimate partner violence and mental health. The *European Journal of Psychotraumatology* has been growing, continuously contributing to the body of knowledge pertaining to mental health and psychology. We are honored to present this special issue of the *European Journal of Psychotraumatology*. The combination of intimate partner violence and psychological trauma is a very common occurrence and to target one of these problems implies managing both (Campbell, 2002). Thus far, little attention has been paid to intimate partner violence apart from the psycho-physiological aspect that results (Depierro, D'Andrea, & Pole, 2013).

Intimate partner violence is highly prevalent globally, which presents worrisome implications for mental health. As explained by the World Health Organization, women who are abused have higher rates of both suicidal ideation and suicide attempts than other women (World Health Organization [WHO], 2011). Our previous research has found that one in six women presenting to an orthopedic trauma clinic had a history of physical abuse and one in 50 women presented to a fracture clinic as a direct result of intimate partner violence–related injuries (PRAISE Investigators, 2013). These numbers speak directly of physical injuries and serve as a window into the probable psychological complications associated with these injuries.

In the past, societies often enacted legislation that enabled and even encouraged violence against women, and ergo we are still feeling the impact of past mindsets. It has not been an easy progression, and to this day we still see women struggling against a notion that has been ingrained so deeply into custom. Through research initiatives and communities such as those fostered by the *European Journal of Psychotraumatology*, we can work together towards the ultimate goal of reducing intimate partner violence, and subsequent psychological trauma, to the point of elimination. This issue of the *European Journal of Psychotraumatology* presents important research which has an overarching focus on intimate partner violence and its impact on mental health outcomes for the woman herself, her children, and her family. The research presented in this issue further explores the characteristics associated with abused women, including addictions, mental health outcomes, and the impact such violence has on children. Furthermore, this cluster brings you research on intimate partner violence of indigenous women and how it impacts their communities. The *European Journal of Psychotraumatology* encourages the proliferation of knowledge, and it is with such knowledge that we can create effective change. The journal encourages you to submit your research to build a stronger foundation for better clinical care and for further research.

This cluster brings you seven articles, each being a further step in understanding the link between intimate partner violence and mental health outcomes. “Assessing the co-occurrence of intimate partner violence domains across the life-course: Relating typologies to mental health” describes factors that are most likely to breed intergenerational transmission of violence (Armour & Sleath, 2014). The authors examine the influence of life-course polyvictimization, witnessing parental victimization, and psychological victimization on perpetration of intimate partner violence. Life-course polyvictimization was highly significant in the perpetration of violence. These results provide an indication of the factors that need to be addressed in order to effectively reduce further propagation of violence.

Child abuse, may it be physical or emotional, has been a trigger leading to the victim becoming a perpetrator. As studied in “Childhood maltreatment and intimate partner violence in dissociative disorder patients,” childhood maltreatment often leads to dissociation, which is later manifested as dissociative disorders (Webermann, Brand, & Chasson, 2014). Physical and emotional abuse experienced in childhood is associated with perpetrating physical intimate partner violence later on in life while neglect in childhood is associated with perpetrating emotional intimate partner violence. This unfortunately lends to a cycle that is not easily broken.

Violence has negative ramifications on mental health; however, “Adult experience of mental health outcomes as a result of intimate partner violence victimisation: A systematic review” reports that the effects of psychological violence on mental health were more prominent than originally thought (Armour, Lagdon, & Stringer, 2014). This review looks at the outcomes of intimate partner violence on mental health and found that the most significant outcomes were depression, post-traumatic stress disorder, and anxiety. These authors also report that the severity of the outcome is dependent on the severity of the abuse.

Intimate partner violence and mental health outcomes are not secluded to a single jurisdiction and women's experiences around the globe share similarity. “Predictors of change in mental health and distress among women attending a women's shelter,” based in Canada, focused on the mental health predictors of women who attended the shelter system more than once (Hoyeck, Madden, Freeman, Scott, & Bhandari, 2014). Increased attendance to a women's shelter increased the likelihood of mental health improvements, providing evidence of the important role shelters play in helping women. In addition, “Intimate partner violence and drug-addicted women: From explicative models to gender-oriented treatments” offers an overview of substance abuse and intimate partner violence, both from a European standpoint and on a globalized level (Simonelli, Pasquali, & De Palo, 2014). This study aims to explain the relationship between intimate partner violence, the given environment, and substance abuse all the while providing a multidisciplinary approach to the problem.

Providing possible solutions always provokes thought and further ideas about how we can help to alleviate these detrimental outcomes. “Effects of an intervention program for female victims of intimate partner violence on psychological symptoms and perceived social support” evaluates a three-phase intervention program for female victims with the purpose of increasing perceived social support (Hansen, Eriksen, & Elklit, 2014). This study reports that the program was effective in reducing psychological symptoms and increasing levels of perceived social support.

Finally, “‘It will always continue unless we can change something’: Consequences of intimate partner violence for indigenous women, children, and families” has a special focus on violence against indigenous women and girls (Burnette & Cannon, 2014). The perspectives of US indigenous women on the impacts of violence were gathered in which the women reported profound psychological consequences due to intimate partner violence. Many had witnessed intimate partner violence as children, further lending weight to the fierce cycle propagating violence from generation to generation. Intimate partner violence is a social issue with dire consequences and targeting this issue is not possible without struggle. There is not one solution but so many variables that need to be considered in this complex equation.

*Sheila Sprague*
Division of Orthopaedic Surgery
Department of Surgery
McMaster University
Hamilton, Ontario
Canada*Miranda Olff*
Department of Psychiatry, Academic Medical Centre
University of Amsterdam &
Arq Psychotrauma Expert Group
Diemen, the Netherlands
